# TSH cut off point based on depression in hypothyroid patients

**DOI:** 10.1186/s12888-017-1478-9

**Published:** 2017-09-07

**Authors:** A Talaei, N Rafee, F Rafei, A Chehrei

**Affiliations:** 1 Department of Internal Medicine, School of Medicine, Endocrinology and Metabolism Research Center, Arak University of Medical Sciences, Arak, Iran; 2 Amiralmomenin Hospital, Arak, Iran

**Keywords:** TSH, Cut off, Hypothyroid, Depression

## Abstract

**Background:**

The prevalence of depressive symptoms in hypothyroidism is high. Considering that hypothyroidism and depression share some clinical features, some researchers use the “brain hypothyroidism” hypothesis to explain the pathogenesis of depression. We aimed to detect a new TSH cut-off value in hypothyroidism based on depression symptoms.

**Methods:**

A cross-sectional study was conducted on hypothyroid patients referred to endocrine clinics. Individuals who had developed euthyroid state under treatment with levothyroxine with TSH levels of 0.5–5 MIU/L with no need for dosage change were included in the study. After comprehensive history taking, laboratory tests including TSH, T4 and T3 were performed. Beck depression questionnaire was completed for all patients by trained interviewers. TSH cut-off values based on depression was determined by Roc Curve analysis.

**Results:**

The participants were 174 hypothyroid patients (Female; 116: 66.7%, Male; 58: 33.3%) with mean age 45.5 ± 11.7 (19–68) years old. Based on Beck depression test, scores less than 10 was considered healthy and more than 10 were considered depressed. According to Roc curve analysis, the optimal cut- off value of TSH was 2.5 MIU/L with 89.66% sensitivity. The optimal TSH cut- off based on severe depression was 4 MIU/L.

**Conclusion:**

The present study suggests that a clinically helpful TSH cut-off value for hypothyroidism should be based on associated symptoms, not just in population studies. Based on the assessment of depression, our study concludes that a TSH cutofff value of 2.5 MIU/L is optimal.

## Background

Thyroid hormones play a significant role in brain development. Thyroid hormone deficiency during pregnancy can lead to delayed neuropsychiatric syndrome and cretinism. Thyroid hormones exert various effects on different processes such as neurogenesis, glial development, myelination, synaptogenesis, and dendritic cell proliferation. Therefore, these hormones are essential for normal brain development and function ([1–3]). The relationship between brain and thyroid was first reported by Parry in 1786 [4]. Furthermore, in 1873, Gull showed the association between psychosis and myxedema [5].

The prevalence of depressive symptoms in hypothyroidism has been reported to be 60% [6], while the prevalence of clinical hypothyroidism in psychiatric individuals has been estimated at 0.5–8% [7]. Forgetfulness, fatigue, mental slowness, and emotional lability have been shown as some psychological symptoms of hypothyroidism. It seems, there is no correlation between the existence and severity of psychiatric symptoms and the degree of thyroid hormone abnormalities.These psychological symptoms can severely compromise one’s quality of life [8].

According to a study by Yu, subclinical hypothyroidism, following Graves’ disease treatment, could increase the incidence of depression symptoms. Also, the severity of depression symptoms was found to be correlated with the level of serum thyroid stimulating hormone (TSH). In addition, levothyroxine replacement therapy could lead to the symptomatic improvement of depression [9]. Hypothyroidism and depression are similar in terms of clinical features.

Therefore, some researchers have used the “brain hypothyroidism” hypothesis to explain the pathogenesis of depression. In this theory, depression is a state of local hypothyroidism in the brain with normal peripheral thyroid hormone level as a result of deiodinase type II inhibition and impaired transport of T4 across the blood-brain barrier [10]. This theory is in accordance with the theory of serotonin deficiency in depression [11].

Abnormalities in the hypothalamus-pituitary-thyroid (HPT) axis have been reported in people with depression. In fact, several studies have confirmed the existence of classical feedback between the serotonergic and HPT systems. Thyrotropin-releasing hormone (TRH) is constantly inhibited by serotonin. On the other hand, reduced intracerebral serotonin concentration in depression could lead to increased TRH concentration in brain tissues. This mechanism is probably responsible for the blunted TSH response to TRH stimulation in depression [10]. In animal studies, thyroid hormones influence noradrenergic and serotonergic neurotransmission, which is a target for current antidepressant therapies and plays a key role in the pathogenesis of depression [12].

Use of thyroid hormone for depression treatment has been controversial for a long time. For most practitioners, the normal reference range for TSH seems to be the most challenging issue in detecting thyroid problems and monitoring the effectiveness of treatment. Nevertheless, there are still unresolved disagreements about the standard upper limit of TSH [13]. Wartofsky showed, a new TSH range was needed.

The findings indicated the absence of an absolute cut-off value for TSH to distinguish between normal and abnormal; the mean normal TSH level ranged only between 1.18 and 1.4 mU/L. Also, the fact that more than 95% of the normal population had a TSH level below 2.5 mU/L could support the assumption that an individual with a higher TSH level should be assessed for thyroid dysfunction [13].

A TSH level within the laboratory reference range is not necessarily normal for every individual. Some evidences suggest that one single TSH reference range does not fit the entire population [14]. To the best of our knowledge, no TSH cut-off value has been established for depression diagnosis. In hypothyroid patients treated with levothyroxine, psychological symptoms, especially depression, may persist even when they achieve a euthyroid state ([15]). With this background in mind, we propose a new TSH cut-off value as a guideline to evaluate and treat depression symptoms.

## Methods

This cross-sectional study was performed on 174 hypothyroid patients, aged 19–68 years. The subjects were patients referred to endocrine clinics, who had developed a euthyroid state under levothyroxine treatment (Euthyrox, Merck Company) with a TSH level of 0.5–5 mU/L over the past year and no need for dosage change. The patients had no underlying diseases and did not use any other medications. After comprehensive history taking and physical examniation, laboratory tests including TSH, T4, and T3 were performed. TT4 and TT3 measurements were performed, using the radioimmunoassay (RIA) method.

In addition, TSH level was measured by the immunoenzymometric assay (IRMA), using commercial kits (Izotop, Budapest, Hungary) and gamma counters (Wallac Wizard, Wallac Oy, and Turku, Finland). Intra- and inter-assay coefficients of variation (CV) were 3.3% and 6.2% for TT4, 6.7 and 7.8% for TT3, and 3.9% and 7.1% for TSH, respectively. Depression was evaluated based on Beck Depression Inventory (BDI), Beck and Mendelson version in 1961 that revised in 1986. The BDI was completed for all patients by trained interviewers. In general, BDI consists of 21 questions, with responses ranging from “no symptoms” (0 points) to “severe symptoms” (3 points).

This questionnaire was analyzed in terms of reliability 76 and validity, and a Cronbach’s alpha coefficient (internal consistency) of 0.87 was obtained. In addition, the reliability coefficient of repeated test was 0.79 after a week and the correlation of the score of each question with the score of other questions of the questionnaire and also with the total score of the questionnaire was meaningful [16]. In this study, MMPID(Minnesota Multiphasic Personality Inventory-Depresion) scale was used for reliability of BDI. The data were analyzed using MedCalc software, and TSH cut-off value was determined by receiver operating characteristic (ROC) curve analysis. This study was conducted in accordance with the Declaration of Helsinki. Written consent forms were obtained from all the subjects.

## Results

**Fig. 1:**
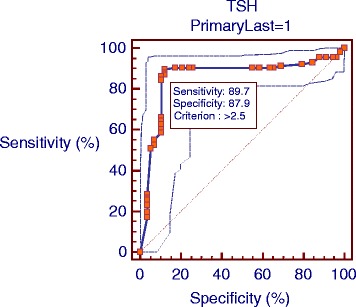
Area under curve for TSH cut off based on depression

The study population was composed of 174 hypothyroid patients (female: 116, 66.7%; Male: 58, 33.3%) under treatment with levothyroxine and mean ages of 45.5 ± 11.7 years (range: 19–68 years). The patients were evaluated in terms of depression by BDI. Based on this questionnaire, scores below 10 were considered as normal, while scores above 10 were indicative of depression. As the findings revealed, 116 subjects were depressed, while 58 cases were healthy. The mean for T4 and T3 were 8.4 pg/dl and 1.2 ng/ml respectively. Different cut-off points were compared to detect the optimal cut-off value for TSH. Based on the ROC curve analysis, TSH cut-off point of 2.5 mU/L showed 89.66% sensitivity and 87.93% specificity, and with the Area Under Curve (AUC) 86% was the optimal cut-off point for depression diagnosis (Fig. 1 & Table 1). In addition, the positive predictive value (PPV), negative predictive value (NPV), positive likelihood ratio (PLR), and negative likelihood ratio (NLR) of different TSH cut-off points were determined and compared (Table 2).

**Table 1:** Sensitivity and specificity for different TSH (MIU/L) cut-off points based on depression (ROC analysis)

TSH cut off (MIU/L)	Sensitivity (%)	CI 95% for sensitivity		Specificity (%)	CI 95% for specificity	
		Lower	Upper		Lower	Upper
2	90.52	83.7	95.2	74.14	61	84.7
2.5*	89.66	82.6	94.5	87.93	76.7	95
3	66.38	57	74.5	89.66	78.8	96.1

**Table 2:** PPV, NPV, PLR, and NLR for different TSH cut-off points based on depression (ROC analysis)

TSH (MIU/L)	PLR	CI 95% for PLR		NLR	CI = 95% for NLR		PPV (%)	CI = 95% for PPV		NPV (%)	CI = 95% for NPV	
		Lower	Upper		Lower	Upper		Lower	Upper		Lower	Upper
2	3.5	3	4.1	0.13	0.06	0.3	87.5	80.2	92.2	79.6	66.5	89.4
2.5*	7.43	6.6	8.3	0.12	0.05	0.3	93.7	87.4	97.4	81	69.1	89.8
3	6.41	5.5	7.5	0.37	0.2	0.8	92.8	84.9	97.3	57.1	46.3	67.5

**Fig. 2:**
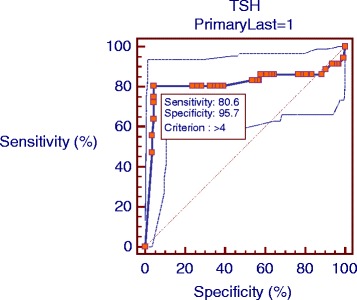
Area under Roc curve for TSH cut off based on severe depression

Then among patients with severe depression (BDI score > 19), different TSH cut-off points were determined and compared based on severe depression. According to the ROC curve analysis, the optimal TSH cut-off point was 4 mU/L with respect to severe depression (Fig. 2 & Table 3). In addition, PPV, NPV, PLR, and NLR of different TSH cut-off points were determined and compared based on severe depression (Table 4).

**Table 3:** Sensitivity and specificity for different TSH (MIU/L) cut off points based on severe depression (ROC analysis)

TSH	Sensitivity (%)	CI = 95% for sensitivity		specificity (%)	CI = 95% for specificity	
(MIU/L)		lower	upper		lower	upper
3.5	80.56	64	91.8	65.94	57.4	73.8
4	80.56	64	91.8	95.65	90.8	98.4
4.5	63.89	46.2	79.2	95.65	90.8	98.4

**Table 4:** PPV, NPV, PLR, and NLR for different TSH cut-off based on severe depression (ROC analysis)

TSH (MIU/L)	PLR	CI 95% for PLR		NLR	CI 95% for NLR		PPV(%)	CI = 95% for PPV		NPV(%)	CI = 95% for NPV	
		Lower	Upper		Lower	Upper		Lower	Upper		Lower	Upper
3.5	2.37	1.9	2.9	0.29	0.1	0.6	38.2	27.2	50.1	92.9	85.8	97.1
4*	18.53	15.7	21.8	0.2	0.07	0.6	82.9	66.1	93.6	95	89.9	98
4.5	14.69	11.5	18.8	0.38	0.2	0.9	79.3	59.9	92.2	91	85.2	95.1

## Discussion

The present study showed a significant correlation between the BDI score and TSH and T4 levels. Also, the findings showed that a TSH cut-off point of 2.5 mU/L is the optimal cut-off value for considering depression symptoms and treatment. To the best of our knowledge, this is the first study to focus on TSH cut-off point for depression. Many patients in spite of being euthyroid present with symptoms such as sleep disorders, fatigue, and depression in particular ([15]). These symptoms might be related to deiodinase II [17] or brain thyroid hormones transporter genes such as organic anion-transporting polypeptide I (OATP-C1) polymorphisms [18].

The relationship between hypothyroidism and depression has been confirmed in previous literature [19]. In fact, depression is the most common neuropsychiatric symptom in hypothyroidism [20]. It seems that there are analogous mechanisms leading to the similarity of symptoms in hypothyroidism and depression [19]. In addition, depression is associated with changes in the HPT axis [21]. Since 1960, many physicians have used thyroid hormones for the treatment of depression [22]. Zhang et al. revealed reduced serotonin levels in the dorsal raphe nucleus (DRN), resulting from inhibition by an overactive 128 lateral habenula (LHb) in depressed hypothyroid rats. It was concluded that LHb could mediate the effect of thyroid system on serotonin function in DRN [23].

According to the literature, 63.5% of cases with subclinical hypothyroidism have depression, and levothyroxine treatment does not lead to the full recovery of the symptoms [24]. Although different TSH cut-off points have been introduced for the treatment of hypothyroidism in population-based studies, persistence of hypothyroidism symptoms in many patients, despite of being euthyroid, indicates the need for a symptoms-based TSH cut-off point [16]. Obviously, the best TSH cut-off point should be based on all thyroid clinical functions and symptoms including depression, which is one of the most significant symptoms.

The TSH cut-off point suggested in our study is based on the patients’ depression score on BDI. The TSH cut-off point of 2.5 mU/L showed 89.7% sensitivity and 87.9% specificity, which seems more suitable in comparison with 2 and 3 mU/L cut-off points. Also, PPV of 93.7% and NPV of 81% indicate the superiority of this cut-off value over other cut-off points. In other words, a TSH cut-off point of 2.5 mU/L can accurately identify 93.7% of depressed patients (based on BDI score) and rule out depression in 81% of cases. Also, PLR of 8.3% shows that depressed people in comparison with hypothyroid cases without depression are eight times more likely to have a TSH level above 2.5 mU/L. Also, an NLR of 0.12 shows that a TSH level below 2.5 mU/L is 0.1 times more probable in depressed individuals in comparison with hypothyroid cases without depression; therefore, this cut-off value was more appropriate for identifying depressed subjects, compared to other cut-off points.

It seems that a TSH cut-off point of 4 mU/L (with 80.5% sensitivity and 95.6% specificity) is optimal for identifying severe depression in comparison with 3.5 and 4.5 mU/L values. In fact, this cut-off point could detect 82.9% of severely depressed cases. Also, regarding to PLR, a TSH level above 4 mU/L was 21 times less likely 153 in severely depressed individuals, compared to cases without depression. Also, the chance to have TSH level below 4 in severely depressed people in comparison to subjects without depression is just 0.2. Although studies with the aim to identify the optimal TSH cut-off point are often population-based, comparison of the findings suggests that a TSH cut-off point with respect to depression is in accordance to them.

In fact, various studies have introduced different population-based cut-off points in individuals without any clinical or serological evidences of thyroid disease. TSH cut-off points were determined to be 3.4, 0.6–3.7, 0.2–4.6, 0.38–4.2, 0.4–3.4, 0.3–5.1, 1.3–3.1, 0.39–4.2, and 0.7–7 mU/L in studies by Zarcovic [25], Chan in Hong Kong (on Chinese cases) [26], Rosario in Brazil (on aged subjects) [27], Kutluturk in Turkey [28], Langen in Finland [29], Sriphrapradang in Thailand [30], Marwaha in adult Indians [31], Yoshihara in Japan [32], and Kim in Korea [33], respectively. Nevertheless, Haddad in Texas could not detect a TSH cut-off point and found that a lower TSH level could lead to the relief of fatigue [34]. Kutluturk recommended that a population-specific TSH level should be detected [28]. Marwaha concluded that a TSH cut-off point should be age- and gender-specific [31]. Also, in the study by Yoshihara on aged Japanese subjects, the upper limit of the normal range for serum TSH increased with age [32].

In several studies, no association could be found between depression and TSH levels, and there was no need to reduce the upper reference limit for TSH level [35]. As mentioned earlier, no TSH reference range can fit all ages, races, or sexes. Therefore, it is important to consider various factors in determining a TSH cut-off value. The present results could provide a depression-specific cut-off point for TSH, which may help improve patient care. Of course there are some limitations due to small sample size, convenience recruitment and also the population of the study was not large enough to provide the statistical power to analyze the effects of sex or age and there is the need for broad based studies.

## Conclusion

The present study identify a TSH value of 2.5 mU/L as the optimal cut-off point to predict depression symptoms in hypothyroid patients treated with T4.

## Abbreviations

AOR: Adjusted odds ratio
CGPA: Cumulative grade point average
COR: Crude Odds Ratio
ETB: Ethiopian Birr
IOT: Institute of Technology
MMPID: Minnesota Multiphasic Personality Inventory-Depresion
NGO: Non Government Organization
SPSS: Statistical Package for Social Science
WHO: World Health Organization

## References

[1] Hetzel BS, Chavadej J, Potter BJ (1988). The brain in iodine deficiency. Neuropathol Appl Neurobiol.

[2] Zoeller RT, Rovet J (2004). Timing of thyroid hormone action in the developing brain: clinical observations and experimental findings. Neuroendocrinology.

[3] Williams GR (2008). Neurodevelopmental and neurophysiological actions of thyroid hormone. J Neuroendocrinol.

[4] Esposito S, Prange AJ, Golden RN (1997). The thyroid Axis and mood disorders: overview and future prospects. Psychopharmacol Bull.

[5] Jackson IMD, Asamoah EO (1999). Thyroid function in clinical depression: insights and uncertainties. Thyroid Today.

[6] Bathla M, Singh M, Relan P (2016). Prevalence of anxiety and depressive symptoms among patients with hypothyroidism. Indian J Endocrinol Metab.

[7] Radhakrishnan R, Calvin S, Singh JK, Thomas B, Srinivasan K (2013). Thyroid dysfunction in major psychiatric disorders in a hospital based sample. Indian J Med Res.

[8] Vigário Pdos S, Vaisman F, Coeli CM, Ward L, Graf H, Carvalho G (2013). Inadequate levothyroxine replacement for primary hypothyroidism is associated with poor health-related quality of life-a Brazilian multicentre study. Endocrine.

[9] Yu J, Tian AJ, Yuan X, Cheng XX (2016). Subclinical hypothyroidism after 131I-treatment of Graves' disease: a risk factor for depression. PLoS One.

[10] Foltyn W, Nowakowska-Zajdel E, Danikiewicz A, Brodziak A (2002). Hypothalamic-pituitary-thyroid axis in depression. Psychiatr Pol.

[11] -Jacobsen JP, Medvedev IO, Caron MG. The 5-HT deficiency theory of depression. Philos Trans R Soc Lond B Biol Sci.2012; 367(1601): 2444–2459. 10.1098/rstb.2012.0109 PMC3405680 22826344

[12] Mason GA, Bondy SC, Nemeroff CB, Walker CH, Prange AJ (1987). The effects of thyroid state on beta-adrenergic and serotonergic receptors in rat brain. Psychoneuroendocrinology.

[13] Wartofsky L, Dickey RA (2005). The evidence for a narrower thyrotropin reference range is compelling. J Clin Endocrinol Metab.

[14] Biondi B (2013). The normal TSH reference range: what has changed in the last decade?. J Clin Endocrinol Metab.

[15] Saravanan P, Chau WF, Roberts N, Vedhara K, Greenwood R, Dayan CM (2002). Psychological well-being in patients on 'adequate' doses of l-thyroxine: results of a large, controlled community-based questionnaire study. Clin Endocrinol.

[16] Rajabi G, Atari Y, Haghighi J (2001). Factor analysis of Beek depression inventory (II) among male student of Chamran University in Ahvaz. J Educ Psychol.

[17] Panicker V, Saravanan P, Vaidya B, Evans J, Hattersley AT, Frayling TM (2009). Common variation in the DIO2 gene predicts baseline psychological well-being and response to combination thyroxine plus triiodothyronine therapy in hypothyroid patients. J Clin Endocrinol Metab.

[18] van der Deure WM, Appelhof BC, Peeters RP, Wiersinga WM, Wekking EM, Huyser J (2008). Polymorphisms in the brain-specific thyroid hormone transporter OATP1C1 are associated with fatigue and depression in hypothyroid patients. Clin Endocrinol.

[19] Dayan CM, Panicker V (2013). Hypothyroidism and depression. Eur Thyroid J.

[20] Ingram E, The international encyclopedia of depression, 2009; Springer, New Yorka.

[21] Kirkegaard C, Faber J. The role of thyroid hormones in depression. Eur J Endocrinol. 1998;138:1-9. 10.1530/eje.0.1380001 9461307

[22] Prange AJ, Wilson IC, Rabon AM, Lipton MA (1969). Enhancement of imipramine antidepressant activity by thyroid hormone. Am J Psychiatry.

[23] Zhang Q, Feng JJ, Yang S, Liu XF, Li JC, Zhao H (2016). Lateral habenula as a link between thyroid and serotoninergic system modiates depressive symptoms in hypothyroidism rats. Brain Res Bull.

[24] Demartini B, Masu A, Scarone S, Pontiroli AE, Gambini O (2010). Prevalence of depression in patients affected by subclinical hypothyroidism. Panminerva Med.

[25] Žarković M, Ćirić J, Beleslin B, Ćirić S, Bulat P, Topalov D (2011). Further studies on delineating thyroid-stimulating hormone (TSH) reference range. Horm Metab Res.

[26] Chan A, Iu YP, Shek CC (2011). The reference interval of thyroid-stimulating hormone in Hong Kong Chinese. J Clin Pathol.

[27] Rosario PW, Calsolari MR (2014). TSH reference range in older adults: a Brazilian study. Arq Bras Endocrinol Metabol.

[28] Kutluturk F, Yildirim B, Ozturk B, Ozyurt H, Bekar U, Sahin S (2014). The reference intervals of thyroid stimulating hormone in healthy individuals with normal levels of serum free thyroxine and without sonographic pathologies. Endocr Res.

[29] Langén VL, Niiranen TJ, Mäki J, Sundvall J, Jula AM (2014). Thyroid-stimulating hormone reference range and factors affecting it in a nationwide random sample. Clin Chem Lab Med.

[30] Sriphrapradang C, Pavarangkoon S, Jongjaroenprasert W, Chailurkit LO, Ongphiphadhanakul B, Aekplakorn W (2014). Reference ranges of serum TSH, FT4 and thyroid autoantibodies in the Thai population: the national health examination survey. Clin Endocrinol.

[31] Marwaha RK, Tandon N, Ganie MA, Mehan N, Sastry A, Garg MK (2013). Reference range of thyroid function (FT3, FT4 and TSH) among Indian adults. Clin Biochem.

[32] Yoshihara A, Noh JY, Ohye H, Sato S, Sekiya K, Kosuga Y, Suzuki M (2011). Reference limits for serum thyrotropin in a Japanese population. Endocr J.

[33] Kim M, Kim TY, Kim SH, Lee Y, Park SY, Kim HD (2015). Reference interval for thyrotropin in a ultrasonography screened Korean population. Korean J Intern Med.

[34] El-Haddad B, El Bizri I, Kallail KJ, Zackula RE, Hoffman JM (2012). Fatigue and TSH levels in hypothyroid patients. Kansas Journal of Medicine.

[35] Eskelinen SI, Vahlberg TJ, Isoaho RE, Löppönen MK, Kivelä SL, Irjala KM (2007). Associations of thyroid-stimulating hormone and free thyroxine concentrations with health and life satisfaction in elderly adults. Endocr Pract.

